# Barriers to pilot mobile teleophthalmology in a rural hospital in Southern Malawi

**DOI:** 10.11604/pamj.2014.19.136.5196

**Published:** 2014-10-09

**Authors:** Guillermo Martínez Pérez, Wayne Swart, Jimmy Kondwani Munyenyembe, Peter Saranchuk

**Affiliations:** 1 Southern Africa Medical Unit, Médecins Sans Frontières, Cape Town, Southern Africa; 2 Department of Mechanics and Mechatronics, University of Stellenbosch, South Africa; 3 Médecins Sans Frontières, Malawi

**Keywords:** mHealth, HIV/AIDS, Malawi, opportunistic infections, eye diseases, mteleophthalmology

## Abstract

**Introduction:**

Malawi has one of the highest HIV prevalences in Sub-Saharan Africa. The rate of eligible HIV-infected people being initiated on antiretroviral therapy (ART) and retained in HIV-care is currently far from adequate. Consequently, many people continue present with advanced immunosuppression at public health facilities, often with undiagnosed opportunistic infections (OIs).

**Methods:**

In this context, mHealth was the innovation chosen to assist Eye Clinical Officers in early diagnosis of HIV-related diseases having eye manifestations in a rural hospital in Thyolo, Southern Malawi.

**Results:**

The mTeleophthalmology program began in October 2013, but was stopped prematurely due to organizational and technological barriers that compromised its feasibility.

**Conclusion:**

Sharing these barriers might be useful to inform the design of similar innovations in other resource-limited settings with a high HIV prevalence and a dearth of eye specialists with capacity to diagnose HIV-related retinopathies.

## Introduction

Malawi, a landlocked country in east Africa, has one of the highest HIV prevalences in the world, estimated at 10.6% in 2010. By the end of 2012, UNAIDS estimated that approximately 1,100,100 Malawians were living with HIV [1]. According to Ministry of Health (MoH) HIV/AIDS guidelines, all HIV-infected patients that present with a CD4 count result below 350 cells/µl, as well as all pregnant and lactating women, regardless of their CD4 count, should be initiated on antiretroviral therapy (ART) [2]. Nevertheless, the reality is that many HIV-infected people eligible for ART have not yet been initiated on it. Moreover, among those Malawians ever initiated on ART, a significant proportion have either become lost-to-follow-up or, for a wide variety of reasons, have discontinued their treatment and are no longer receiving HIV-care [1, 3].

This low uptake of MoH HIV/AIDS guidelines means that many people continue to present at public health facilities with symptoms of severe immunosuppression. There have been difficulties getting an accurate epidemiological picture of the incidence and characteristics of the different opportunistic infections (OIs) among the HIV-infected population in Malawi [4]. Some OIs can be difficult to diagnose, especially in resource-limited settings; in such cases, clinical examination of the eye can assist in diagnosis, since certain OIs can have eye manifestations (e.g. disseminated tuberculosis, toxoplasmosis, and cytomegalovirus) [5, 6]. Early initiation of ART, retention of patients in HIV-care, and training clinicians, including Eye Clinical Officers (ECOs) to diagnose and treat these OIs early can prevent vision loss and other morbidity, plus mortality [5–7].

**mHealth**, innovative technological solutions hold the potential to assist in early diagnosis of HIV-related OIs with eye manifestations. An information and communication technology that is burgeoning in low-income countries is mobile health (mHealth). A broadly accepted standard definition of mHealth is ‘medical and public health practice supported by mobile devices, such as mobile phones, patient monitoring devices, personal digital assistants (PDAs), and other wireless devices’ [8]. Specific to the context in Malawi, Group Special Mobile Association (GSMA), an association of mobile operators and related companies, has identified the increase in access to mobile network geographic coverage, widespread use of mobile sharing, and increasingly higher deployment of short message services (SMS), unstructured supplementary service data (USSD), and interactive voice response (IVR) services as good opportunities for demand-creation of mHealth [9]. Globally, there is a growing emerging market for Smartphone applications (‘apps’) to capture, store, compress and transmit images taken by mobile built-in or wirelessly synchronized diagnostic tools. The potential for state-of-the-art, mobile-enabled monitoring and diagnostic tools is vast and includes, for instance, microscopes to identify helminthic eggs [10], electrocardiogram (ECG) readers [11], or urine tests for ovarian cancer biomarkers [12]. All these information and communication technologies and these mobile diagnostic software advances are worth being capitalized in resource-constrained settings such as rural Malawi.

**mTeleophthalmology** attempts to trial teleophthalmology programs in other high HIV-prevalent countries have been documented in the scientific peer-review literature: a teleglaucoma initiative has been piloted in Kenya [13] and teleophthalmology aimed to improve diagnosis of diabetic retinopathies in Cameroon [14]. However, mHealth was not the approach used to design the abovementioned examples of teleophthalmology programmes. In Malawi, with the purpose being to help diagnose HIV-related diseases with eye manifestations, a mobile-based teleophthalmology program (hereafter mTeleophthalmology) was introduced in October 2013 in Thyolo District Hospital (TDH). To our knowledge, no specific mobile-based teleophthalmology program to diagnose HIV-related OIs with eye manifestations has ever been piloted in east Africa. mTeleophthalmology was designed as an innovation with the potential to build capacity of ECOs in fundoscopic examination and clinical management of HIV-related retinopathies. The aim of this article is to describe, as lessons learnt from the field, the organizational and technological barriers that impeded the implementation of mTeleophthalmology beyond its pilot phase. A secondary aim is to suggest use of these findings to inform future initiatives in other contexts characterized by similar constraints.

## Methods

### Study site

mTeleophthalmology was designed as operational research in TDH, a tertiary health facility in Thyolo District, Southern Malawi. TDH covers a population of approximately 600,000 of which 14% are estimated to be HIV-infected [3]. The primary research objective was to assess feasibility of mTeleophthalmology to build ECO's capacity in fundoscopic examination and enable earlier diagnosis of HIV-related OIs with eye manifestations. The hospital is administered and staffed by MoH. At the time of the pilot, TDH had an Eye Clinic staffed by only one ECO. The Eye Clinic received programmatic and logistic support from the medical humanitarian organization Médecins Sans Frontières (MSF).

### Materials

All HIV-infected patients with eye manifestations that presented at the TDH Eye Clinic were offered to be enrolled as study participants. If consent was given, images of the fundus of both eyes were captured using the camera from an iPhone 4S (TMApple Inc, Cupertino, CA) attached to a PanOptic ophthalmoscope (TMWelch Allyn, New York, NY). The PanOptic ophthalmoscope allows the clinician a panoramic view of the fundus, including the optic nerve, through an undilated pupil; a theoretical field of view (FOV) of up to 25o can be achieved, which is 5 times larger than the standard 5o FOV using a traditional direct ophthalmoscope. To capture the images, the iExaminer ‘app’ (TMWelch Allyn, New York, NY) was installed on the iPhone 4S. iExaminer is mobile software that allows clinicians to capture, store and retrieve images taken with the PanOptic, in association with a basic set of patient data. The iExaminer ‘app’ works offline in a store-and-forward mode; images can be sent once access to a Wi-Fi hub has been enabled, such that a local SIM card and a contract with a mobile network operator are not necessary. The next step involved sending the images to remote eye specialists for review, which required that a comprehensive dataset of clinical information be associated for each case. Thus, instead of using the iExaminer to enter patients’ information, it was opted to use Collegium Telemedicus, a MSF web-based messaging system that links specialist advice through e-referral to health personnel working in resource-limited settings. Two ophthalmologists based in California (USA) and in Western Cape (South Africa) respectively reviewed the e-referrals during the pilot phase of the program.

### Ethics

All eligible patients were informed of the purpose of the research. Participants gave written informed consent for the ECO to capture images of their fundi and to transmit the images, together with anonymized clinical information, to the two remote ophthalmologists. It was explained to the patients that the main harm they could undergo as a consequence of participating in the research was associated with the discomfort caused by the eye drops used to dilate their pupils. Only data that related to their eye examination (e.g. visual acuity test) and to their HIV-infection process (specifically, last CD4 count result and viral load (VL) count, ART regimen and date of initiation, and previous or current history of OIs and other non-HIV diseases) were shared with the ophthalmologists. No personal identifiers were ever transmitted through Collegium Telemedicus, so there was no risk of disclosure of HIV status. A fictitious Identification Number was created to link the e-referral case with the patients’ clinical chart that was stored in a locked cabinet at the TDH Eye Clinic. The study protocol was approved by National Health Science Research Council (Lilongwe, Malawi, Approval Number NHSRC#1205) and by the Médecins Sans Frontières Ethical Review Board (Geneva, Switzerland).

## Results

mTeleophthalmology started at the Eye Clinic in October 2013. The decision to discontinue the program was officially communicated to Malawi's National Health Sciences Research Committee (NHSRC) four months later. During that period, sixteen HIV-infected patients were recruited as study participants. In the paragraphs below, the main barriers that impeded implementation of mTeleophthalmology beyond its pilot phase are described.

### Organizational barriers

The Eye Clinical Officer (ECO), a medical assistant (MA) trained in basic eye care, had limited capacity to identify abnormal fundoscopy findings, since the curriculum of MAs in Malawi does not include comprehensive training on diagnosis of retinopathies. In addition, this ECO did not have a solid foundation in computer skills. A mHealth Officer from MSF was required to assist the Eye Clinic staff in the rather arduous task of capturing, storing, labeling, and transmitting images in the format of an e-referral and for which a predetermined amount of clinical information had to be included. Limited human resources represented another organizational barrier: an Eye Clinic staffed by a single ECO in a rural tertiary hospital that provides assistance to a population of approximately 600,000 people is undoubtedly not the most suitable working environment to dedicate the time required to strictly comply with the standard operating procedures (SOPs) involved in this operational research. To capture good quality images for a single patient using the PanOptic-iExaminer-iPhone combination, it could take 45 minutes or more of the ECO's time. This time increased for patients who had difficulty cooperating, for example by blinking and moving their heads because of the discomfort they were experiencing due to the illumination of the scope. The PanOptic-iExaminer-iPhone combination was not perceived as a time-efficient point-of-care diagnostic procedure. The Eye Clinic personnel were preoccupied by the patients queuing to enter the consultation room. As per operational research procedures, the ECO was expected to conduct regular ward rounds in the Tuberculosis, HIV and Kaposi Sarcoma Units to recruit potential study participants among the HIV-infected inpatients having eye complaints or whose latest CD4 count was below 100 cells/µl. The workload at the Eye Clinic made it difficult for the ECO to comply with this assigned task.

Other organizational barriers were intrinsic to information management constraints of the health system in Malawi. Capturing real-time, accurate clinical data for patients was a major challenge. There is no district-wide electronic medical record (EMR); instead, the hospital was using an EMR system since 2013 called Baobab [15], into which information on HIV-infected patients accessing HIV-care at TDH was being entered. But patients arriving from remote villages did not have a personal record in Baobab. In many cases, either their HIV-related data (VL, CD4 count, and ART regimen) was not documented in their personal paper ART cards, or they had traveled to the Eye Clinic having left their card at home. This lack of personal data complicated the completeness and quality of the e-referrals, and, ultimately, made it more difficult to arrive at a conclusive diagnosis.

### Technological barriers

It was the technological barriers that ultimately determined that mTeleophthalmology was not feasible in this setting. In essence, the only devices that the ECO needed were the PanOptic ophthalmoscope, an iPhone 4S and the iExaminer software. Smooth technical implementation was prevented because of several issues: the first was proprietary licensing, as the ‘Pro version’ of the iExaminer application, required to print and transmit images via email from the phone, is not freeware and is not available for purchase via iTunes Store in Malawi. Secondly, interoperability was a barrier, since there were perceived to be too many steps to be followed by the ECO in order to transmit the fundoscopic images from the iPhone 4S to a laptop in the Eye Clinic using the Windows operating system and on to Collegium Telemedicus through a GSM modem. Thirdly, market forces made it difficult to find the 4S version of iPhone in stores following the arrival of the ‘state-of-the-art’ iPhone 5 in Malawi. Fourthly, network coverage was a problem around the time of the pilot phase, GPRS and 3G networks were frequently either interrupted or very weak to transmit a set of six jpeg-compressed images in a single try.

**Figure 1:**
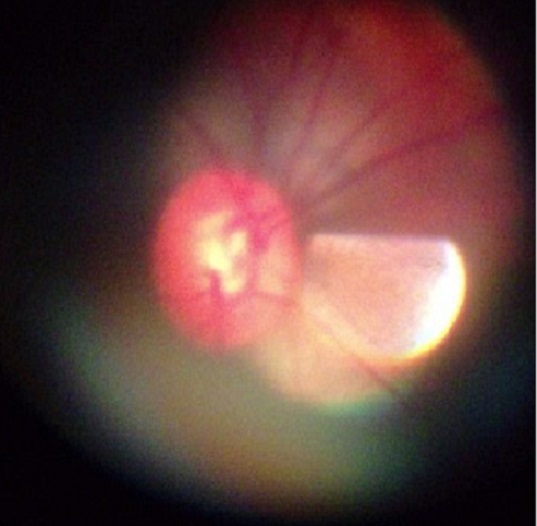
Most images had artifacts or reflections

**Figure 2:**
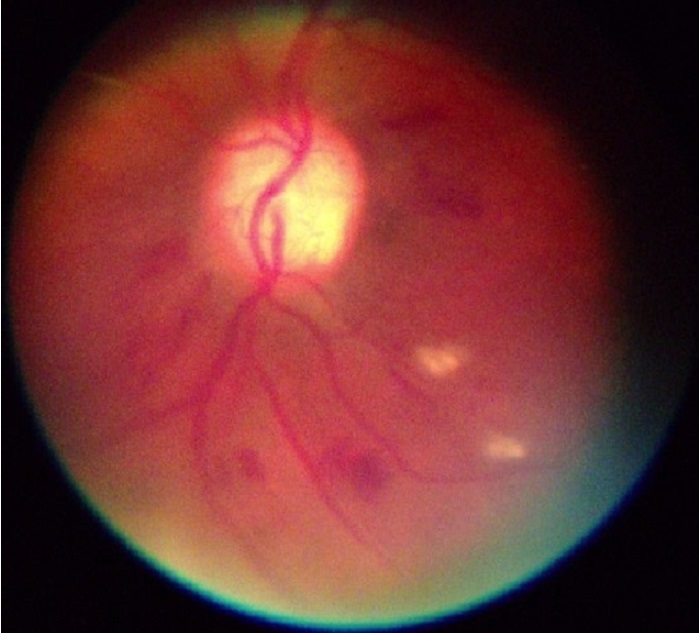
Most images were blurred in the periphery

Fifthly, and most importantly, though retinal pathology was seen in some images, the majority of e-referrals were left unresolved because the quality of the images captured with the PanOptic-iExaminer-iPhone combination was not satisfactory enough as a stand-alone diagnostic tool to allow the remote ophthalmologists to reach a conclusive diagnosis with confidence. Although the patient eyecup on the PanOptic instrument is meant to screen out ambient light, and the glare extinguishment system expected to help prevent interference from unwanted glare and reflections, there were internal reflections or artefact in most images (Figure 1). Moreover, even if the images were of good quality in the middle, they were blurred in the periphery because of the lens design of the PanOptic (optical aberration) (Figure 2). These issues can be attributed to the fact that the PanOptic was designed for fundus-to-eye use rather than as a fundus-to-camera interface.

**Figure 3:**
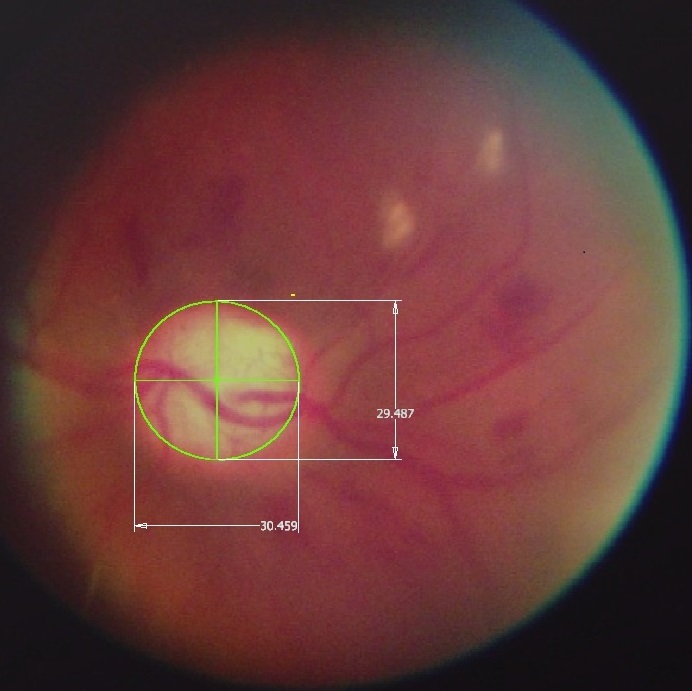
Measurement of the optic disc

The FOV performance of a fundus imaging system can be determined by measuring the optic disc on the captured images and comparing the measurements to biometric data from literature (Figure 3) [16]. The PanOptic-iExaminer-iPhone combination captured images with a FOV between 4.5 mm and 7.5 mm of the fundus such as those shown in ( Figure 1, Figure 2) respectively. These dimensions included the blurred regions on the periphery and thus the FOV performance of the PanOptic-based camera is relatively poor compared to more established commercial systems that can capture up to 13.5 mm of the fundus without any blur on the periphery, which is the range most ophthalmologists are familiar with and indeed required for a definitive diagnosis. Despite the need for the study ophthalmologists to have perspectives from the four different quadrants of the fundus, because it was time-consuming for the ECO to capture good images, all e-referrals contained images from only one of the quadrants.

## Discussion

Organizational and technological barriers compromised implementation of mTeleophthalmology in a rural tertiary hospital in Southern Malawi and local stakeholders perceived the innovation as unfeasible. The decision to discontinue the program was based on the evident need to address health system deficiencies, including its EMR system and other basic technological requirements, before considering re-initiation of this initiative at a later stage with a fundoscopic camera that takes better quality images. Should the decision be taken to re-launch mTeleophthalmology in this setting, an ethnography-informed human-computer interaction approach might be a useful methodological choice to re-design the implementation process and to define SOPs that can make a technologically complex such as this usable, intuitive and acceptable to the local health personnel [17, 18].

It is necessary to involve district delegates of the MoH at the inception of a program of this caliber. An iterative design and planning process with the MoH as principal stakeholder is crucial in order to align the expected benefits of mTeleophthalmology with the strategic vision of their HIV/AIDS programmes activities that aim to provide capacity building for clinicians working in decentralized HIV-care settings, early diagnosis of OIs, together with quality HIV-care and treatment. As well, it is important to address information gaps around existing patient-level health information systems. MoH-driven changes in human resources are necessary to strengthen capacity in the Eye Clinic before any future mHealth (or other) innovations can be considered feasible. Not only do additional human resources need to be allocated to this specific unit, but the staff also need to have a good foundation in computer and smartphone skills, in order to ensure that work processes and patient flow are optimal. The MoH can also play an invaluable role by incorporating into the ECO's job description tasks that relate to routine mTeleophthalmology, as well as by advocating for improvements in university curricula of medical assistants on routine fundoscopic examination, and diagnosis and treatment of retinopathies. With regards to the technological barriers identified, it is necessary to improve the interoperability of devices and systems. Procuring hardware manufactured by companies that adhere to international device communication interoperability standards, such as CEN ISO/IEEE 11073 [19], must be studied as a sustainable solution for resource-limited settings.

Based on our experience, we have yet to find an ophthalmoscope that can take quality fundoscopic images in health facilities in rural and resource-limited settings; features that need to be improved in the PanOptic-iExaminer-iPhone combination that we tested include the field of vision, and lenses that can focus across all margins of the images, as well as to minimize the discomfort created to patients caused by the lack of hybrid internal illumination. With respect to transmission of images, Android-based (open) systems are preferable, for interoperability and sustainability purposes, rather than systems that depend on the iPhone operating system. Future research on mTeleophthalmology should test the design, functionalities, and the quality of images taken by the PanOptic-iExaminer-iPhone combination against other ophthalmoscopes supported by fundoscopic photography solutions that become available in the market. However, a limitation of our experience with mTeleophthalmology is that the innovation was piloted in the frame of an operational research. The burden of the research-related standard operating procedures in the daily routine activities at the Eye Clinic might have contributed to this innovation being perceived as unfeasible. Collaboration with MoH in the design of new attempts of implement telemedicine in low-resourced settings, should these attempts be planned as research, is crucial to minimize or eradicate all pre-identified human and organizational barriers.

## Conclusion

Although human resources and a suboptimal imaging device were major barriers in our attempt to pilot mTeleophthalmology in a rural hospital in Southern Malawi, this innovation should still be considered as a useful approach to build clinicians’ capacity on fundoscopic examination, ensure quality and continuity of HIV-care, and prevent permanent vision loss and other morbidity among HIV-infected people in sub-Saharan Africa. HIV-related morbidity could be decreased if early and accurate diagnosis and treatment of opportunistic infections having eye manifestations were possible at the primary care level, by an experienced clinician, and with distance support from an ophthalmologist [6, 7]. Unexpected and non-anticipated barriers, together with proposed solutions, must be an essential element in the design of any telemedicine plan; however, this may be difficult to achieve due to the dearth of evidence-based literature of similar experiences in sub-Saharan African. Hence, lessons learnt such as the ones we are sharing in this article must be disseminated among the scholarly community to inform the design and planning of future initiatives that aim to facilitate early diagnosis and treatment of any HIV-related conditions having eye manifestations.
